# Affections of the Eye Caused by Masturbation

**Published:** 1881-06-20

**Authors:** M. Landesberg


### AFFECTIONS OF THE EYE CAUSED BY MASTURBATION.

BY M. LANDESBERG, M. D.

The relation of masturbation to diseases of the eye has scarcely attracted the attention of the profession. In the best hand-books of ophthalmology there is no reference to this subject, and in the ophthalmic literature, as far as my knowledge goes, this fact is mentioned but twice only. Dieu (see Nagel's Jahresberichte der Ophthalmologie, 1872, p. 372) records the case of a boy of five years in whom amblyopia developed in consequence of masturbation. After the removal of the existing congenital phimosis, which was the exciting cause of the self-pollution, the latter was given up, and vision gradually improved to normal condition. Fœrster (see Handbuch der gesammten Augenheilkunde, von Græfe and Sæmich, V. vii., part V., p. 102) has witnessed instances of intractable chronic catarrh of the eye in patients of from twelve to twenty years, in whom onanism was ascertained to be the only cause of the affection. For my part, I have reason to assume that chronic inflammations of the eye, resulting from masturbation, are not of such rare occurrence as we might be led to infer from the scarcity of the published material on this subject. I remember having met in my practice with many cases of obstinate catarrhal affections of the eye which I had to give up in despair after a protracted course of unavailing treatment, or the patients left me in order to seek better advice. At the time, I was at a loss to account for the intractableness of such cases. Catarrhal affections of the eye generally give a good prognosis, and are easily cured if properly attended to. I had to yield to the evidence that there are some forms of affection of the conjunctiva in which the treatment fails to bring about the usual beneficial effect. These forms I generally met with in children of either sex, but occasionally, also, in adults. When I afterward learned the intimate relation that exists between some morbid processes of the eye and masturbation, there was no doubt left to me about the nature of all those intractable cases which have been so mortifying to the self-confidence of the physician. This opinion was corroborated by the many other indications of self-pollution which I had observed in these patients, and the pathognomonic symptoms of which I utterly disregarded for want of the proper knowledge of this peculiar coincidence.

The first case that gave me the key to the problem, was a merchant, aged thirty-three years, who came to me suffering from chronic catarrh of both eyes. He had been for nine months under the care of a prominent oculist, who had tried every available remedy without any result. There were no anomalies of refraction or accommodation. Both eyes showed only the symptoms of chronic catarrh with slight blepharitis. The affection had lasted about a year. No reasonable cause of the morbid process could be elicited. There was no inflammation of the other mucous membranes. General health was good. The patient was in good circumstances, and temperate in his habit of drinking and smoking. He was very anxious to get rid of his trouble, and was willing to undergo any treatment for this purpose. I must say I was not a little astonished at the failure of the previous treatment, the traces of which (slight argyria) were seen on both eyes. I made a good prognosis, and promised a perfect cure.

In the course of the treatment, I was struck by the observation that the improvement I succeeded in bringing about in the condition of the eyes did not remain steady, but was interrupted by frequent exacerbations of the morbid process. For a long while I was baffled in all my efforts to find any plausible explanation of this strange incident. One day, when my patient came to me with a renewed relapse, it occurred to me that the pimples he had on his face were much more inflamed, and more numerous than on the preceding days. On further observation, I ascertained, beyond any doubt, that the increase of the inflammation and number of the pimples always coincided with the deterioration of the morbid process of the eye. The connection of pimples of the face with masturbation, I had frequent occasion to establish in either sex. I was roused to the suspicion whether the anomalous affection of the conjunctiva might not depend altogether upon masturbation. I inquired of the patient concerning his habits in regard to the other sex. He told me that, for the last eighteen months from the time he had incurred a gonorrhœa, he has discontinued all sexual intercourse with women. On further inquiry he confessed that from that time he has been masturbating about two or three times a week. The pimples of his face developed consequently. He has, also, observed that after masturbation, the condition of his face and of his eyes becomes worse. This coincidence impressed his mind so strongly, that he had spoken with his family physician about it, but the latter had derided any possibility of such a relation.

I imparted to him my conviction that onanism has been in his case the only cause of his eye affection, and that no cure could be effected unless the habit was totally abandoned.

The patient being of a resolute nature, at once discontinued the practice, and had the satisfaction of seeing his eyes gradually improve, without any further treatment whatever. In the course of a month, all traces of the inflammation vanished, and the face became smooth and fair.

From this occurence I made a point to inquire in every instance of intractable catarrh of the eye, after this possible error of youth. I learned from experience that it is very difficult to find out the truth in this matter in the male sex, but that it is almost impossible to ascertain it in the female one.

